# Smart Glasses for Older Adults With Cognitive Impairment: Explanatory Mixed Methods Study

**DOI:** 10.2196/81840

**Published:** 2026-04-27

**Authors:** Brittany F Burch, Nayeon Kim, Rachel McPherson, Debora Anokye, Alice S Ryan, Odessa Addison, Nirmalya Roy, Chien-Ming Huang, Elizabeth Galik, Barbara Resnick

**Affiliations:** 1 School of Nursing, University of Maryland Baltimore, 655 W Lombard St, Baltimore, MD, 21201, United States, 1 4107063770 ext 53770; 2 School of Medicine, University of Maryland, Baltimore, MD, United States; 3 Baltimore Geriatric Research, Education, and Clinical Center, VA Maryland Health Care System, Baltimore, MD, United States; 4 Information Systems, University of Maryland, Baltimore, MD, United States; 5 Whiting School of Engineering, Johns Hopkins University, Baltimore, MD, United States

**Keywords:** technology, cognitive impairment, smart glasses, dementia, older adults

## Abstract

**Background:**

Smart glasses might present a promising solution to support older adults with cognitive impairment in maintaining independence. However, there exists a critical gap in smart glasses research that incorporates recently developed models or directly engages older adults with cognitive impairment.

**Objective:**

This study aimed to use survey and interview techniques to explore the acceptability and usability of smart glasses among older adults with cognitive impairment.

**Methods:**

This explanatory mixed methods descriptive study was conducted at an independent living older adult apartment building among residents with memory complaints or cognitive impairment. This study consisted of a quantitative survey (N=26), followed by smart glasses beta testing with qualitative interviews (n=14).

**Results:**

Overall, older participants with cognitive impairment conveyed a generally positive perception of smart glasses and their potential to support memory in daily life. Results suggest that participants prioritized the following smart glasses functions: audio reminders, phone calls, GPS, and distress signals, with audio reminders emerging as the highest-ranked feature. Additionally, participants emphasized the value of an intuitive and quick interface and a preference for audio, rather than visual, information exchange.

**Conclusions:**

This study supports the development and study of smart glasses for older adults with mild cognitive impairment. Smart glasses developers should place great importance on usability, such as intuitive command interfaces, and equip the smart glasses with functions that are relevant to this population, especially audio reminders. Additionally, future work should examine the integration of smart glasses over a longer period and among a larger sample of older adults with cognitive impairment.

## Introduction

### Older Adults With Cognitive Impairment: Prevalence and Burden

Cognitive impairment is a condition associated with a decline in one or more cognitive functions such as memory, attention, reasoning, language, problem-solving, and decision-making [1-3]. The severity of cognitive impairment can range from mild cognitive impairment to more advanced conditions, including severe dementia. In mild cognitive impairment, individuals might struggle with complex tasks such as managing medications and making phone calls, but more basic self-care tasks remain largely preserved [3,4]. The global prevalence of cognitive impairment among community-dwelling older adults is approximately 19%, and this prevalence is expected to rise as the population ages [1,2]. Among older adults who are economically disadvantaged, the onset of cognitive impairment occurs earlier, and the prevalence of cognitive impairment is higher [5,6].

Cognitive impairment is often associated with depression and limitations in activities of daily living, significantly affecting an individual’s quality of life [7]. Caregivers of individuals with cognitive impairment may also experience emotional, physical, or financial strain due to the sequelae of symptoms associated with cognitive impairment [8,9]. While some research indicates that cognitive training, physical activity, and pharmacological interventions can delay the progression of cognitive impairment, no cure currently exists [10-14]. Consequently, innovative solutions are needed to help individuals maintain independence as they live with their cognitive impairment.

### Smart Devices to Assist Older Adults With Cognitive Impairment

Smart devices are defined as context-aware electronic tools that are capable of performing autonomous computing and connecting for data exchange [15]. They present innovative solutions to support older adults with cognitive impairment in maintaining independence. For example, researchers have explored smartphones, smart wristwatches, and tablet-based applications to assist with cognitive training or function as an external memory aid [16-19]. Similarly, researchers have investigated virtual assistant smart speakers, such as Google Home or Amazon Alexa, to provide in-home support to older adults living in the community with cognitive impairment [20,21]. While such technologies demonstrate promise in supporting older adults with cognitive impairment in living independently, common acceptability and usability barriers include nonintuitive interfaces, a lack of personalization and accessibility, and technology literacy limitations among older adults [16,20,21]. Over the past decade, a new type of smart device—smart glasses—has emerged as a potential tool to assist individuals with memory-related challenges [22]. Unlike other technological interventions, glasses are a familiar and commonly worn accessory among older adults, potentially making smart glasses a more natural and accepted aid.

### The History of Smart Glasses

For the purposes of this paper, smart glasses are defined as an eye-worn device that provides functions similar to a computer to assist and augment the wearer [23]. The release of the Google Glass Explorer Edition from 2013 to 2014 marked the first consumer-grade smart glasses available to the public. However, issues such as unattractive designs, privacy concerns, and high cost led to its limited adoption, and the product was discontinued in 2015 for purchase by general consumers [24-26]. In 2017 and 2019, Google released updated versions, Google Glass Enterprise Edition and Google Glass Enterprise Edition 2, marketed more specifically to businesses; these were discontinued in 2023 [27]. Around this time, the first models of mixed reality headsets were also released, such as the Microsoft HoloLens (2016) [28] and the Magic Leap One (2018) [29]. While at the forefront of innovation in fields such as health care, manufacturing, education, and defense, these devices were still largely considered niche industry products and not marketed to or adopted by general consumers [28-31]. Additionally, their bulky design and lack of portability raise questions about whether the devices should be classified as “smart glasses”—in addition to a headset [22].

In the last 5 years, advancements in features, esthetic, and affordability have led to the growing integration of smart glasses into everyday life, especially among younger individuals [32]. Current models incorporate advanced technologies, including cameras, microphones, GPS, and accelerometers, while maintaining the appearance of regular eyewear [22]. These features enable functionalities such as voice assistance, navigation, reminders, photo live or live stream, and social connection, provided in a more discreet manner. Of note, some commercial smart glasses are becoming powered with advanced artificial intelligence (AI; eg, Ray-Ban Meta) and augmented reality technology (eg, Vuzix V100), defined as digital information overlaid onto the real environment [33]. Given the recent technological and esthetic improvements of commercial smart glasses, alongside the increasing population burden of cognitive impairment, further research is necessary to explore their acceptability and usability among older adults.

### Smart Glasses Research and Gap

To contextualize this topic, our research team recently conducted a systematic scoping review on smart glasses for older adults with cognitive impairment [22]. Among the 13 studies identified, only 5 [34-38] involved older adults in hands-on testing of the smart glasses, and only 1 study [37] exclusively recruited older adults living with cognitive impairment, though it was limited by a small sample size (n=6). Additionally, all included studies in the review focused on earlier generations of commercial smart glasses, such as Google Glass and HoloLens, or early-stage rudimentary prototypes (ie, regular glasses with components added to the side arm of the glasses frames). Our review underscores a critical gap in research that incorporates recently developed smart glasses models and directly engages older adults with cognitive impairment. To address this gap, this study aimed to use survey and interview techniques to explore the acceptability and usability of smart glasses among older adults with cognitive impairment. For the purposes of this paper, and consistent with the Sekhon Theoretical Framework of Acceptability, we define acceptability as “the extent to which people delivering or receiving a health care intervention consider it to be appropriate, based on anticipated or experienced cognitive and emotional responses to the intervention” [39]. Next, consistent with the International Organization for Standardization (ISO 9241‐11), we define usability as “the extent to which a system, product, or service can be used by specified users to achieve specified goals with effectiveness, efficiency, and satisfaction in a specified context of use” [40]. The results from this study will guide researchers in selecting appropriate smart glasses and guide engineers in developing smart glasses that are more accessible and relevant for this population across both research studies and real-world settings.

## Methods

### Study Design

This was an explanatory mixed methods descriptive study to explore the acceptability and usability of smart glasses among older adults with cognitive impairment and consisted of two phases: (1) a quantitative survey and (2) smart glasses beta testing with qualitative interviews.

### Participants

Given the high prevalence and burden of cognitive impairment among economically disadvantaged older adults [5], we recruited participants from an income-controlled independent living older adult apartment building in Baltimore. We recruited participants through flyers posted in the apartment building and a brief on-site presentation about the project. Residents were eligible if they met the following criteria: (1) scored 4 or lower on the Mini-Cog [41] or 3 or higher on the Memory Complaint Scale [42], and (2) passed the Evaluation to Sign Consent Measure [43], which assessed whether participants understood the study purpose, risks, and withdrawal rights. As we specifically invited residents who perceived themselves as having memory problems or forgetfulness, nearly all residents (26/27) who expressed interest in the study were eligible based on the cognitive screening criteria. For phase 1 (surveys), we enrolled all interested residents who met the eligibility criteria. For phase 2 (glasses testing and interviews), we randomly selected phase 1 participants for interviews until we achieved data saturation, defined as redundancy of data with no newly emerged themes [44].

### Ethical Considerations

The University of Maryland, Baltimore, Institutional Review Board approved the protocol for this study (HP-00109956). We informed participants of the study procedures, risks, benefits, and withdrawal rights prior to participation. We protected confidentiality by assigning unique study IDs to participants and storing all identifiable data on secure, password-protected servers and in a locked office within a locked cabinet. Participants provided informed consent prior to participation. Participants received a US $30 incentive for completing the survey and interview, respectively.

### Procedures

During phase 1, we administered a paper-based quantitative survey, which we read aloud and completed for participants upon request. Once phase 1 was completed for all participants, we purchased 3 different pairs of smart glasses that best aligned with participants’ preferences and priorities, as informed by the survey feedback: Ray-Ban Meta smart glasses, Alexa Echo Frames, and Vuzix Blade 2 smart glasses. The 3 pairs of selected smart glasses have many similarities, such as including a speaker and a microphone. One primary distinction is that the Alexa Echo Frames do not have a camera, whereas the other 2 devices do. Additionally, Vuzix Blade 2 smart glasses provide augmented reality functionality, which is not available in the other 2 smart glasses.

During phase 2, a random sample of participants from phase 1 beta-tested these 3 pairs of smart glasses and provided qualitative interview feedback. During beta testing, each participant completed a task list for each pair of glasses, guided by the research assistant, as detailed in Textbox 1. Glasses testing took approximately 20 minutes per pair, for a total of 60 minutes. Participants tested the glasses pairs in a rotating order to minimize order effects. For example, participant 1 tested Ray-Ban Meta smart glasses first, Alexa Echo Frames second, and Vuzix Blade 2 smart glasses third. Alternatively, participant 2 tested Alexa Echo Frames first, Vuzix Blade 2 smart glasses second, and Ray-Ban Meta smart glasses third.

Textbox 1. **Participants’ beta-testing task list.**

**Ray-Ban Meta smart glasses**

- “Hey Meta, how is the weather outside?”
- “Hey Meta, remember for my shopping list I’ll need eggs, milk, and bread.”
- “Hey Meta, play [insert favorite music].”
- “Hey Meta, remember that I put my purse/wallet by the door.”
- “Hey Meta, remember this book.”
- “Hey Meta, remind me in 2 minutes to take my medications.”
- “Hey Meta, where is the closest Safeway to me?”
- “Hey Meta, what street am I on?”
- “Hey Meta, look and tell me what this says in English?”
- “Hey Meta, what items are on my shopping list?”
- “Hey Meta, look at this snack and tell me if it’s healthy.”
- “Hey Meta, look at this cooking tool and tell me what it is called.”
- “Hey Meta, look at this recipe and summarize it. Hey Meta, remind me what I do after microwaving the butter.”
- “Hey Meta, take a photo now and send it to [research assistant].”
- “Hey Meta, call [research assistant].”
- “Hey Meta, take a picture of this flyer so I can refer to it later.”
- “Hey Meta, remind me where I put my purse today. What was the book I wanted to remember?”
- “Hey Meta, play ‘Mindfulness for Beginners’ on the Calm app.”

**Alexa Echo Frames**

- “Hey Alexa, how is the weather outside?”
- “Hey Alexa, play [insert favorite music].”
- “Hey Alexa, remind me in 2 minutes to take my medications.”
- “Hey Alexa, where is the closest Safeway to me?”
- “Hey Alexa, what is the metal tool you use to beat eggs?”
- “Hey Alexa, what items are on my shopping list?”
- “Hey Alexa, are raisins healthy?”
- “Hey Alexa, call [research assistant].”
- “Hey Alexa, what’s on my ‘to do’ list?”
- Participant demonstrates the “find my” function within app (GPS tracking).
- “Hey Alexa, turn off lights.”

**Vuzix Blade 2 smart glasses**

- Scroll through apps with voice: “Hey Vuzix. Move right. Move left.”
- Scroll through apps with fingers.
- Select notifications with fingers.
- Select notifications with voice: “Hey Vuzix, notifications.”
- Determine preferred method (audio or voice).
- Take a picture with preferred method.
- Take a video with preferred method.
- Navigate to Microsoft Teams and check calendar with preferred method.
- Make a call with preferred method.

### Measures

#### Phase 1

The phase 1 quantitative self-report survey consisted of three sections, which assessed (1) demographics, including age, gender, living alone status, comorbidities, and financial strain; (2) frequency of technology use; and (3) preferred smart glasses functions. To assess the frequency of technology usage, we administered the FACETS (Functional Assessment of Currently Employed Technology Scale) [45], a validated and reliable 10-item questionnaire that measures the frequency of technology use across the following areas: sending emails, accessing files, sending text messages, posting on social media, online banking, online purchases, telehealth, communicating with health insurance online, installing computer components, and resetting a modem or router [45]. We removed the last 2 items, which were less pertinent to those who live in the congregate affordable housing setting, many of whom primarily use smartphones and public building Wi-Fi. Item responses ranged from 0 (“never”) to 5 (“daily/prefer”). Thus, total scores ranged from 0 to 40, with higher scores indicating greater technology usage.

As this work is exploratory, no validated survey currently exists that examines the preferred functions of smart glasses. Instead, we developed the “preferred functions” items based on our clinical expertise with this population and dementia-friendly survey guidelines from the Alzheimer’s Society [46]. This section contained 17 Likert-style questions in which participants rated smart glasses’ potential functions as “not helpful,” “slightly helpful,” “very helpful,” and “most helpful.” Participants then reported the 3 “best” smart glasses functions from all listed functions. The survey is provided in Multimedia Appendix 1.

#### Phase 2

Immediately following the testing of each of the three pairs of glasses, the participants answered five open-ended questions: (1) What did you like best about these glasses? (2) What did you like least about these glasses? (3) What function did you like best for these glasses? (4) What function did you like least for these glasses? (5) Is there anything else you would like to share about these glasses? Upon completing beta testing for all 3 pairs, participants responded to five additional overarching questions: (1) What did you think of the smart glasses overall? (2) Describe the glasses you liked the best and why. (3) Describe the smart glasses functions you liked the best and why. (4) Are there any functions you wish smart glasses could have that these did not? (5) Why or why not do you think smart glasses could be used to help with memory? We intended the questions about participants’ likes, dislikes, and overall impressions to capture acceptability, while the questions about specific functions, ease of use, and desired features were intended to capture usability. To maintain consistency, a single interviewer (BFB) with qualitative interviewing expertise conducted all interviews. The interviewer provided assistance as needed, while avoiding leading questions or comments. The interviewer transcribed the feedback verbatim in real time.

### Analysis

After collecting phase 1 quantitative surveys, we conducted descriptive statistics and applied data visualization approaches to summarize the quantitative results. Specifically, we obtained mean FACETS results and ranked participants’ preferred smart glasses functions based on how frequently each was identified as “the best function” or placed among the top three functions.

One study researcher (BFB) conducted a content analysis to organize and interpret the qualitative data. This process consisted of (1) codebook creation, (2) familiarization with the data, (3) data organization, and (4) data interpretation. First, BFB created the codebook in a deductive manner, based on the topics and questions in the interview guide, reflecting the study aims. This codebook contained the codename, when the code should be used, when the code should not be used, and an example corresponding quotation. Second, and consistent with content analysis, BFB engaged in data familiarization by reading each interview twice. Third, BFB used MaxQDA (VERBI Software), a qualitative data management software, to organize and match segments of text with codes that aligned with them. This coding process was iterative in nature. After coding the first 2 interviews, BFB refined the codebook to capture the advantages and disadvantages of each pair of smart glasses with greater granularity. The finalized code names, subcode names, and their frequency of use are listed in Multimedia Appendix 2. Finally, following the methods outlined by Merriam and Tisdell [47], codes were interpreted so that those with similar concepts were merged into categories, and categories were further integrated into themes.

Once the themes were identified and summarized, a second researcher (NK) independently familiarized herself with the qualitative interview data for the purpose of peer-debriefing. During peer debriefing, BFB and NK met to engage in reflexive dialogue, assess for any themes not expressed within the results, consider alternative interpretations of the data (rival explanations), or identify data that did not align with our current conclusions (negative cases). This collaborative process was conducted to enhance the trustworthiness and credibility of our results. While the peer debriefing did not result in the addition of any new themes, it served as the catalyst for a more detailed explanation of participants’ preferred smart glasses functions.

The research team then met to compare survey data results with interview findings, identifying convergent, complementary, and divergent conclusions.

Following triangulation, the research team returned to the older adult apartment building to present results and conduct member checking with available participants (n=18). During this session, participants were asked, as a group, to provide feedback on (1) conclusions that reflected their experience and perspective, (2) conclusions that misrepresented their experience or perspective, and (3) any additional insights that were missing or they wanted to share. In response, participants conveyed that the major conclusions drawn by the research team resonated with their experience and perspective. Of note, 2 small changes were made due to the member checking session; namely, language translation was removed from the list of functions described as least helpful, and facial recognition was added within the “Recommendations for Functions Smart Glasses Should Do” section.

## Results

### Overview

Among the 26 older adult participants, 16 (61.54%) identified as female, 18 (69.23%) identified as Black or African American, and 14 (53.85%) reported some level of financial strain (Table 1). The participants scored an average of 3.50 (SD 1.42) on the Mini-Cog and 7.62 (SD 3.40) on the Memory Complaint Scale, suggesting that participants overall had an average of mild cognitive impairment, although there was a range of cognition from mild to severe cognitive impairment and memory complaints. Participants scored a mean of 27.88 (SD 13.39) on the modified FACETS, demonstrating moderate technology use across most domains [45]. Participants’ frequency of technology use was highest for online banking and SMS text messaging and lowest for social media use and accessing files.

**Table 1.** Participant characteristics (N=26).

Characteristics	Values
Comorbidities, mean (SD)	2.3 (2.06)
Mini-Cog, mean (SD)	3.50 (1.42)
Memory Complaint Scale, mean (SD)	7.62 (3.40)
FACETS^a^, mean (SD)	27.88 (13.39)
Send email	3.35 (2.06)
Find, open, and close files	2.92 (2.19)
SMS text messaging	4.31 (1.98)
Post on social media	2.11 (1.93)
Manage banking online	4.27 (2.13)
Pay bills and make purchases online	4.15 (2.31)
Communicate with doctor online	3.62 (2.26)
Communicate with health insurance company online	3.15 (2.33)
Gender, n (%)	
Female	16 (61.54)
Male	10 (38.46)
Race or ethnicity, n (%)	
Black or African American	18 (69.23)
White	8 (30.77)
Living alone status, n (%)	
Living alone	24 (92.31)
Living with others	2 (7.69)
Age (y), n (%)	
60‐64	4 (15.38)
65‐69	5 (19.23)
70‐74	6 (23.08)
75‐79	7 (26.92)
≥80	4 (15.38)

a FACETS: Functional Assessment of Currently Employed Technology Scale.

### Quantitative Survey Results

All 26 participants completed the survey with no missing data. Consistently, participants identified audio reminders as the “best” smart glasses function. Specifically, 8 (30.77%) participants ranked audio reminders as the “best” smart glasses function, and 14 (53.85%) participants ranked audio reminders in the top 3 smart glasses functions. Following audio reminders, the following functions were the most valued by participants: phone calls, GPS, so that their loved ones can locate them, and sending distress signals. Conversely, the following functions were reported as least helpful by participants: playing music, cognitive training, subtitles during conversation, companionship and conversation, and providing video or photos for family. All participants (100%) stated that they would be willing to wear smart glasses that assist with memory; however, 6 (23.08%) reported that the glasses *must* look and feel like normal glasses to warrant wear.

### Qualitative Interview Results

#### Overview

A random sample of 14 participants beta-tested 3 pairs of smart glasses: Ray-Ban Meta smart glasses, Alexa Echo Frames, and Vuzix Blade 2 smart glasses. We selected these 3 devices because of their capacity to provide reminders, make calls, and provide GPS functionality, consistent with preferences from the quantitative survey results. After beta testing the 3 pairs of smart glasses, the participants provided qualitative feedback. This feedback is described below, consisting of six themes: (1) perceived advantages and disadvantages of Ray-Ban Meta smart glasses, (2) perceived advantages and disadvantages of Amazon Alexa Echo Frames, (3) perceived advantages and disadvantages of Vuzix Blade 2 smart glasses, (4) preferred smart glasses and functions, (5) broad smart glasses concerns, and (6) recommendations for functions smart glasses should do. Additional participant quotations reflecting the themes can be found in Multimedia Appendix 3.

#### Perceived Advantages and Disadvantages of Ray-Ban Meta Smart Glasses

Participants conveyed the most favorable feedback toward the Ray-Ban Meta smart glasses. These favorable views were due to the following: (1) an intuitive and quick interface, (2) functions that they perceived were relevant to them, and (3) comfort and esthetics. First and foremost, participants overwhelmingly appreciated that the Ray-Ban Meta smart glasses were simple to use and responded clearly, quickly, and precisely to their commands. Participant 4 described:

*They were just real simple. Not a lot involved just speaking the command and it did what it did.*

Second, participants emphasized that the functions provided by the Ray-Ban Meta smart glasses were helpful and relevant to their lives. Features that the participants highlighted as advantages of the Ray-Ban Meta smart glasses included reminder notifications and serving as a memory aid (eg, “Remember where I put my purse”), GPS navigation, phone call functionality, and the inclusion of a portable charging case. Additionally, participants appreciated how the integration of the camera allows the Ray-Ban Meta smart glasses to require less information input than the Amazon Echo Frames. For example, participant 7 described how they “…like how it can take a picture of the book without me saying the name of it…take a picture of the ingredients and it breaking them down.” Finally, participants appreciated the comfort and esthetics of the Ray-Ban Meta smart glasses. They described the glasses as lightweight, attractive, stylish, comfortable to wear, and fitting well. They highlighted the glasses’ resemblance to conventional eyewear.

Although participants generally perceived the Ray-Ban Meta smart glasses as responsive to user commands, compared to the other models tested, 3 participants noted that the interface could still benefit from improvements in accurately detecting user speech. In particular, 1 participant (participant 11), who was edentulous and identified as having an accent, reported needing to repeat the activation command “Hey Meta” multiple times before receiving a response. While the augmented reality of the Vuzix smart glasses was generally viewed unfavorably among the study participants as described below, 2 participants suggested that integrating augmented reality technology into the Ray-Ban Meta smart glasses could produce a pair of glasses with comprehensive functionality.

#### Perceived Advantages and Disadvantages of Amazon Alexa Echo Frames

The participants provided generally favorable feedback on the Amazon Alexa Echo Frames, citing various features that they would appreciate integrating into their daily lives. Many such features overlapped with those of the Ray-Ban Meta smart glasses. For example, participants highlighted the reminder notifications, the virtual assistant for answering questions, and the phone call functionality. Participant 2 stated:

*I liked everything about these glasses. They are right up my alley. The [locating] grocery stores, weather, calling someone, [creating and referring to a] to-do list, locating people.*

Unique to the Amazon Alexa Echo Frames, some participants appreciated how the glasses could sync with other household appliances. In terms of comfort and esthetics, participants generally felt positive toward the lightweight and comfortable frames.

The participants described 3 major disadvantages of the Amazon Alexa Echo Frames. First, some participants emphasized the frequent need to repeat the activation command “Hey Alexa” due to inconsistent voice recognition. Participant 19 explained:

*You have to pause after “Alexa” but then you have to speak fast for what you want. I wish these could do all the things the first one [Meta] could do. You have to repeat.*

Second, participants noted the absence of a camera as a limitation. In addition to the inability to capture photos and videos, the absence of camera restricts the glasses’ capability to perform object recognition or provide information about objects and the environment through visual analysis. Participant 23 stated:

*I like these. But if they had a camera… I wish they had a camera so she could see a lot of things that I want to know.*

Third, participants were concerned that the glasses had a tendency to slide down their nose.

#### Perceived Advantages and Disadvantages of Vuzix Blade 2 Smart Glasses

While feedback on the Vuzix Blade 2 smart glasses was generally negative, some participants described the glasses as interesting and helpful for specific, but limited, situations. Participant 8 stated:

*It was neat having the experience of seeing something inside your glasses [referring to the augmented reality technology]. But that is sort of a novelty thing.*

Participants envisioned the calendar application within Microsoft Teams as a potentially helpful tool for supporting their daily activities. The participants noted that the Vuzix glasses would require significant training and might be more helpful for specific populations, such as individuals with high technological literacy who have hearing or visual impairment.

Among the 3 pairs of smart glasses tested, the Vuzix Blade 2 smart glasses were the least favorably received by participants. The participants expressed three main disadvantages: (1) operational complexity; (2) the distracting nature of the augmented reality interface; and (3) a bulky, unattractive design. First, participants described the Vuzix smart glasses as “complicated,” “strenuous,” and “hard to use.” While the participants appreciated the choice between navigating the glasses through touch and audio input, they noted that the glasses did not respond easily to either input. The touch input was overly sensitive, leading participants to inadvertently navigate to incorrect apps. The touch input (eg, long 2-finger touch to return home) was also difficult for the participants to remember, especially among those with more significant cognitive impairment. On the other hand, while “…Voice commands are more intuitive for people with memory problems” (participant 1), the participants collectively noted that the Vuzix smart glasses had a restricted vocabulary and often failed to respond to their verbal prompts. Next, as the only pair of smart glasses to be equipped with augmented reality technology, participants found the projection on the lens distracting and confusing. Participant 9 stated:

*And the clicking and flashbacks…it’s overwhelming to me. It bothers my eyes. It breaks your concentration. Especially if you’re sitting here talking. It’s flashing and stuff and that bothers me. I’d forget what to do.*

Participants also referred to eye strain and instances of double vision. Finally, there was unanimous agreement that the glasses should be smaller and lighter to improve wearability and transportability. For example, Participant 1 stated:

*I don’t like their look… they are bulky. I mean, what do you put these in? I can’t even put these in my purse. They wouldn’t fit.*

Participants also complained that the glasses fit incorrectly on their faces, such as being heavy on the bridge of the nose, pushing on the head above the ears, sitting on the cheeks, or being tight around the temples.

#### Preferred Smart Glasses and Functions

Following beta testing of the 3 models, 11 participants stated that their preferred glasses were the Ray-Ban Meta smart glasses, 3 participants stated that their preferred glasses were the Amazon Alexa Echo Frames, and no participants stated that their preferred glasses were the Vuzix Blade 2 smart glasses. When asked why they preferred the Ray-Ban Meta smart glasses, the participants overwhelmingly described how the glasses were the “easiest to use.” Participants also mentioned the comfort, esthetics, and camera function in selecting Ray-Ban Meta glasses as their preferred smart glasses. The few participants who preferred the Amazon Alexa Echo Frames smart glasses referred to the lightweight frame.

When asked about the most important functions that smart glasses can have, the participants mainly discussed two features: (1) reminders and (2) instant hands-free information. First, participants found that smart glasses reminders are critical, such as providing alerts to take medications, remembering grocery lists, and remembering where objects are placed. Participant 17 described the most important glasses feature to them:

*For me, to remember things. What was I supposed to get at the grocery store? Where was I supposed to go today? What time was I supposed to get there? Just yesterday I got things messed up and I was supposed to have a procedure and I called just to be safe and they said “You don’t have a procedure today.” So something like this could help prevent these embarrassing moments for someone my age and with my condition.*

The second critical smart glasses function highlighted by participants was instant, hands-free information. Participants stated that smart glasses should provide “clear” and “instant” information through the virtual assistant. Additionally, participants appreciated when the information was delivered through audio feedback, rather than a projection on the lens (augmented reality). Participant 18 described:

*First off you are not holding anything. That is what I like…hands free…to be able to speak out what you need...*

While not endorsed by any other participants, 1 participant (participant 9) emphatically relayed that smart glasses could significantly improve the lives of older adults through offering social companionship for those who experience social isolation. This participant described how older adults could use the glasses for conversation or calling a support group.

#### Broad Smart Glasses Concerns

The discussion related to the Ray-Ban Meta smart glasses and Vuzix Blade 2 smart glasses both elicited privacy concerns among 3 participants. Two participants emphasized the ethical responsibility of glasses users to be respectful of others’ privacy when capturing photos or audio recordings (participants 8 and 23). Participant 3 advocated for more discreet smart glasses designs (compared to Vuzix glasses) to prevent public apprehension about surveillance or invasive behavior. This participant (participant 3) stated that smart glasses could be useful for people with memory problems but said, “You want to make it so, you know, you have the functions, but you don’t want everyone knowing you’re getting the help.” They suggested that a more subtle design would allow for cognitive support without drawing the attention or discomfort of others.

Generally, participants overwhelmingly affirmed that smart glasses could assist with memory by storing information, stirring memory, offering reminders, recalling appointments, aiding with navigation, and recalling where items were placed. Alternatively, 1 among the 14 participants expressed a negative perception of smart glasses’ use for memory within the context of a general overreliance on technology. While this participant (participant 11) believed that smart glasses could be “a potentially useful tool,” they stated:

*I am reluctant to let too much electronics in, myself. I enjoy keeping remembering things myself. Keeping my memory sharp. Whereas with the glasses, it’ll tell me something I don’t necessarily need to know. I’d rather remember in my head and exercise my memory.*

#### Recommendations for Functions Smart Glasses Should Do

After testing the 3 models of smart glasses, the participants provided advice regarding what additional functions smart glasses should provide for older adults who have memory problems. First, participants suggested that smart glasses should identify environmental hazards or dangerous situations, such as recognizing fall risks, dangerous traffic, car accidents, or smoke due to a fire. For example, participant 19 stated:

*They did not point out danger. Like when you got to the corner: “Don’t step down.” “Wait till the light changes.” Help you be more aware of your surroundings.*

Second, participants also stated that smart glasses should provide health assistance, such as step counts or a medical alert in the event of a medical emergency. Third, participants suggested that smart glasses should provide navigation assistance while shopping. For example, smart glasses could group items from a grocery list while navigating the aisles, help locate items in stores, and provide a price check for grocery items. Finally, participants suggested that smart glasses should provide facial recognition for acquaintances. They expressed how a facial recognition functionality would prevent embarrassing moments, such as forgetting the names of other residents in their building.

### Triangulation

The integration of the quantitative and qualitative components of this study revealed convergent and complementary results, strengthening the credibility of the findings. Specifically, both survey and interview results suggest that participants prioritized the following smart glasses functions: audio reminders, phone calls, GPS, and distress signals, with audio reminders emerging as the highest-ranked feature (Table 2). Although participants performed other tasks, such as playing music and sending a photo to family or friends, both the quantitative and qualitative results suggest that these functions were perceived as less essential among older adults with memory problems. In addition to confirming functional priorities, complementary insights emerged from the qualitative interviews. First, following hands-on testing, participants emphasized the value of an intuitive and quick interface. Next, the participants highlighted that audio feedback from the glasses, rather than visual feedback, was the preferred method of information delivery. These complementary qualitative findings reveal that even though certain functions might be perceived as critical, the accessibility of the smart glasses interface is key to participant acceptance.

**Table 2.** Favored smart glasses functions.

Favored functions	Participants who selected the function as the “best” function, n (%)	Itemized survey (1=“least helpful” to 4=“most helpful”), mean (SD)	Example representative quote
Audio reminders	8 (30.77)	3.50 (0.71)	“You know, unless you write it down, you forget it. It can remind you. It’s instant information.” [Participant 3] “Whereas with the others (Meta Ray-Ban smart glasses and Alexa Echo Frames, compared to Vuzix Blade 2), they could function 24/7, you just communicate with it. You don’t have the visual to distract you. I’m one of those people, I can’t multitask, when I’m on my scooter, I have to watch for curbs and people.” [Participant 11]
Phone calls	5 (19.23)	3.38 (0.85)	“Making a call is handy.” [Participant 8] “A lot of the people in this building have family who don’t come see them. These would be beneficial for them. They could sit in their apartment. If they feel lonely or depressed, they could tell the glasses to call a support group or something and it would make the world of difference.” [Participant 9]
Distress signals	3 (11.54)	3.54 (0.71)	“They are going in the right direction with them (smart glasses). If they could do a general programming for them and then customize them to the client’s needs, it would be wonderful. If a person can’t see very well it could analyze for them. It would need sensors to recognize smoke. It should be able to connect to an ambulance like a medical alert…the person’s heart rate and all that good stuff. If the diabetics sugar drops low, it should be able to tell it. It should be able to get it off the phone and help.” [Participant 24] “It would be great if you were stranded…and you could still call someone to save you.” [Participant 2]
GPS	3 (11.54)	3.38 (0.80)	“The question ‘Where am I?’ is a big one…Or if you get lost. These things are important to know.” (Participant 8) “For example, I used to live on [redacted street]. When I moved down here, I traveled all the way to go to Giant. But I could have asked the glasses where the closest Giant is. I find it complicated to look up things on phone and computers and it's easier with the glasses to get information.” (Participant 23)

## Discussion

### Principal Findings

This study used surveys and interviews to explore the usability and acceptability of smart glasses among older adults with cognitive impairment. Both survey and interview results suggest that participants prioritized the following smart glasses functions: audio reminders, phone calls, GPS, and distress signals, with audio reminders emerging as the highest-ranked feature. Following beta testing of 3 pairs of commercial smart glasses, interview results suggest that participants prefer the Ray-Ban Meta smart glasses due to their intuitive and quick interface, comfort and esthetics, and functionally relevant features such as audio reminders and instant hands-free information. Overall, participants conveyed a generally positive perception of smart glasses and their potential to support memory in daily life.

This study supports the development and exploration of smart glasses for older adults with cognitive impairment. Some clinicians might question the foundation of such work, believing that smart glasses for older adults with cognitive impairment could be problematic due to low technology literacy and the potential for increased confusion in this population [48]. As an alternative perspective, this work contributes to the previous literature [49,50], which demonstrates that older adults are diverse in technology use and ability, and many are indeed comfortable with technology and view it favorably. Furthermore, all 26 older participants who participated in this study, including those with lower levels of technology comfort, stated that they would be willing to wear smart glasses that assist with memory. One potential explanation is that the potential benefits for supporting independence could outweigh the expected initial usability challenges. This work demonstrated the strength of user-centered research in emerging fields, allowing individuals to articulate their own preferences and needs.

Although participants conveyed a generally positive perception of smart glasses and their potential to support memory in daily life, they also identified some concerns. For instance, 2 participants highlighted the importance of smart glasses users being respectful of others’ privacy when capturing photo or audio recordings. While enforcing such boundaries could prove practically difficult, most smart glasses have an indicator light that turns on when photo or video is activated to ensure bystanders are aware of such capture. Additionally, 1 participant expressed concern that ongoing use of smart glasses for cognitive assistance could lead to a reliance on the device and a loss of independent cognitive skills. It would indeed be valuable for future longitudinal work to explore whether the cognitive assistance provided by smart glasses in the short term has a “de-training” effect over the long term.

The results of this study suggest that older adults with cognitive impairment strongly value smart glasses that are easy to activate and equipped with a large vocabulary, thus providing interaction that is intuitive. During the past few years, amidst the proliferation of AI, smart glasses have become more user-friendly. As AI technology continues to advance, it is expected that smart glasses will become even more “intelligent,” with improvements in connectivity, data collection and processing, pattern recognition, automation, and adaptability [51]. Over time, as smart glasses require less user input to operate correctly, they could become more appropriate for those with low technology literacy. Going forward, even though the participants verbalized a preference for more polished smart glasses models, older adults with cognitive impairment should still be included in early-stage development research. Including end users in early-stage design of technology helps ensure that the development of technology is applicable and helpful for them and improves the future likelihood of use.

Both survey and interview results suggest that older adults with cognitive impairment perceive audio reminders as the most critical smart glasses function. One of the earliest and most common symptoms of cognitive impairment is episodic memory loss, which is characterized by difficulty in recalling specific personal everyday events and experiences [52]. Audio reminders could help with episodic memory by providing alerts for important upcoming events such as doctor’s appointments, medication administration, or social activities. Similarly, individuals with deficiencies in episodic memory may have difficulty navigating to a new place after 1 visit (eg, a parking garage). These deficiencies are consistent with the participants’ reports of GPS and navigation as prioritized functions.

The participants’ additional preferred smart glasses functions—phone calls and distress signals—reflect the challenges and safety concerns often faced by individuals with cognitive impairment who live alone. Such individuals can experience a sense of uncertainty due to the unpredictable course of symptoms and how these symptoms could negatively impact their lives each day [53]. Especially among this population of economically disadvantaged older adults, many lack adequate home-based services [54]. Smart glasses, equipped with accessible communication features such as phone call capabilities and emergency alerts, may provide older adults with cognitive impairment and their caregivers improved security and assurance that assistance can be readily accessible to the older adult when needed.

### Implications

This work provides guidance for smart glasses developers regarding the design and features that are most important for older adults with cognitive impairment. First and foremost, smart glasses developers should place great importance on usability, including straightforward activation and intuitive command interfaces. Developers should also equip the smart glasses with functions that are relevant to this population, especially audio reminders, and ensure that the glasses are minimalistic so that older adults can receive help in a discreet manner. Finally, and contrary to much current development research [22], until augmented reality technology has considerably advanced, this work suggests that developers of smart glasses for older adults with cognitive impairment should prioritize audio rather than visual feedback for assistance with memory and daily tasks.

This work also provides information for researchers of smart glasses for older adults with cognitive impairment. A recent review on smart glasses for older adults with cognitive impairment, conducted by Burch et al [22], found that the current literature focuses either on outdated commercial smart glasses or rudimentary prototypes, such as regular glasses with bulky technology components (eg, cameras) added to the side arm. This study suggests that current commercial smart glasses provide a promising platform for assisting older adults with cognitive impairment, but additional studies are needed to examine the integration of smart glasses into long-term daily life. Additionally, if health researchers collaborate with computer science researchers on the development of future smart glasses, this work could serve as a strong launching pad for informing preferred smart glasses design and functionality.

Although this work does not explicitly assess the acceptability of smart glasses compared to other smart devices, some interpretive insights can be considered as to why smart glasses might be particularly advantageous. First, participants consistently appreciated the hands-free nature of the smart glasses, which allow users to request information while using a walker or performing a task with both hands. Many other wearable smart devices (eg, smartwatches) are not as supportive of hands-free interaction. Additionally, similar to “earables,” smart glasses are situated near the ear, allowing for more discreet audio assistance than other smart devices. Another reason smart glasses might stand apart compared to other smart devices is that participants valued that the glasses could “see what they see” and provide relevant information about their environment. Finally, participants appreciated that a device they already carry with them could provide such advanced assistance. As smart glasses naturally travel with the user, they provide more possibilities for assistance compared to stationary technologies such as smart speakers. Notably, the aforementioned points should be considered as thought-launching considerations rather than definitive conclusions, as this study was not designed to compare smart glasses with other smart devices.

### Limitations and Strengths

Limitations of this study include a small sample size from a single older adult apartment building, short duration of smart glasses use and testing, and a lack of objective measures. First, the sample size of only 26 participants limits the generalizability of the study results, especially to those with more severe cognitive impairment or those who live in more rural settings. Second, participants beta tested the smart glasses for only 20 minutes per pair. During the testing, the participants could have been influenced by branding, such as associating a particular name brand with quality. Thus, the findings might not translate to long-term preferences and do not reveal information about adherence during everyday life. Finally, no validated survey currently exists that examines the preferred functions of smart glasses. Instead, we developed and used a “preferred functions” survey, which has not been tested for reliability and validity. Responses on this survey during phase 1 informed our selection and purchase of smart glasses for phase 2. As such, participants did not have hands-on experience with the glasses prior to completing the survey.

Study strengths include a user-centered focus, a mixed methods design that included rich interviews, and a sample that reflects older adults who are disproportionately affected by cognitive impairment. Within an emerging field of research, especially those involving new technology, obtaining feedback from intended users is critical to ensure that development and technology selection align well with user needs, thereby increasing the likelihood of adoption. The mixed methodology approach used in this study provided rich insights that numbers alone could not reflect. Finally, we recruited our sample from affordable older adult housing to study a group of older adults who are disproportionately affected by cognitive impairment yet underrepresented in technology research.

### Conclusions

This study aimed to use surveys and interviews to explore the usability and acceptability of smart glasses among older adults with cognitive impairment. Overall, participants conveyed a generally positive perception of smart glasses and their potential to support memory in daily life. Both survey and interview results suggest that participants prioritized the following smart glasses functions: audio reminders, phone calls, GPS, and distress signals, with audio reminders emerging as the highest-ranked feature. Additionally, participants highlighted that audio feedback from the glasses, rather than visual feedback, was the preferred method of information delivery and emphasized the value of an intuitive and quick interface. Future work should examine the integration of smart glasses over a longer period, in a larger sample of older adults with cognitive impairment, and use inferential analytic approaches.

## Supplementary material

10.2196/81840 Multimedia Appendix 1 Survey: preferred smart glasses’ functions.

10.2196/81840 Multimedia Appendix 2 Codebook.

10.2196/81840 Multimedia Appendix 3 Additional participant quotations.

## Abbreviations

AI: artificial intelligence
FACETS: Functional Assessment of Currently Employed Technology Scale
ISO: International Organization for Standardization
