# Supplementary material for: Smart Glasses for Older Adults With Cognitive Impairment: Explanatory Mixed Methods Study
Source: JMIR Aging. 2026 Apr 27;9:e81840. doi: 10.2196/81840 (PMC13119387; doi:10.2196/81840)
Supplement: Multimedia Appendix 1 [file aging-v9-e81840-s001.docx]

**A Survey to Learn Your Preferences for Smart Glasses**

**Why did we make this survey?**

We are a group of nurses from Maryland interested in how smart glasses might improve the quality of life and memory of seniors. We will use the information from these surveys to inform our future work using or adapting smart glasses for seniors.

**What are smart glasses?**

Smart glasses look similar to regular glasses but have some functions that computers have. We want to know what smart glasses’ functions you would dislike, and which you would like.

***Note:** If you wear prescription lenses, assume the smart glasses have the same prescription.

**Please rate the following functions that smart glasses could have from least helpful to most helpful.** *Evaluator: Read through all options on this page first, then go back to the beginning for the ratings.*

|  | Least  Helpful “0” | Slightly Helpful  “1” | Very Helpful  “2” | Most Helpful  “3” |
| --- | --- | --- | --- | --- |
| 1. The glasses can play you music. Example: You can state, “Hey glasses, play Nat King Cole music” and the glasses play you Nat King Cole music. |  |  |  |  |
| 2. The glasses can give you audio reminders throughout the day. Example: You say “Glasses, remind me at 4pm to take my medication.” |  |  |  |  |
| 3. The glasses can provide you with cognitive training to keep your mind sharp. Example: You can state: “Hey glasses, give me a riddle.” |  |  |  |  |
| 4. The glasses can provide you with subtitles when people talk to you. Example: If you have a hearing impairment, the glasses can display on the lens the words that other people are speaking to you. |  |  |  |  |

|  | Least  Helpful “0” | Slightly Helpful  “1” | Very Helpful  “2” | Most Helpful  “3” |
| --- | --- | --- | --- | --- |
| 5. The glasses can provide dietary assistance. Example: You can say to the glasses: “Hey glasses, look at my meal, is consistent with a diabetic diet?” |  |  |  |  |
| 6. The glasses can take notes from conversations. Example: The smart glasses record text/dictation of a conversation so that you can refer to it later if you forget something. |  |  |  |  |
| 7. The glasses can provide speech assistance. Example: If you have a speech impediment or cannot remember a word, the glasses audio will fill in the blanks in your speech out loud. |  |  |  |  |
| 8. The glasses can send a distress signal to your loved ones when you are in trouble. Example: The smart glasses can recognize impact from a fall and send an alert for help to your family. |  |  |  |  |
| 9. The glasses can give you oral education. Example: The glasses can provide audio health information on stroke prevention. |  |  |  |  |
| 10. The glasses can assist you in making phone calls. Example: You can state: “Hey glasses, call my daughter” and you will hear her through the glasses audio. |  |  |  |  |
| 11. The glasses can provide you with map directions. Example: When you are walking to the grocery store, the glasses will orally tell you when and which way to turn and/or the lens will provide arrows when it is time to turn. |  |  |  |  |

|  | Least  Helpful “0” | Slightly Helpful  “1” | Very Helpful  “2” | Most Helpful  “3” |
| --- | --- | --- | --- | --- |
| 12. The glasses can help with monitoring of household devices: Example: You walk out of the home and the glasses state “Did you remember to lock your door?” Later, you can also ask “Hey [glasses], did I turn off the stove?” |  |  |  |  |
| 13. The glasses can provide companionship. Example: The glasses can engage in a conversation about how you are feeling and provide emotional support. |  |  |  |  |
| 14. The glasses can take video recording of what you see or hear *for you*. Example: When you go to the doctor, you can record the visit. Then later, if you forget what your doctor said, you can refer to that video recording. |  |  |  |  |
| 15. The glasses can take a photo for *you* to remember something later. Example: You see a flyer for an event that you want to attend. You take a picture of the flyer with your glasses to refer to it later. |  |  |  |  |
| 16. The glasses can take video and photo of what you see *for your family*. Example: When your loved one wants to check on you, they can take a live photo or video from your glasses of what you see. That photo/video goes directly to their phone. For example, they can see you are safe and watching TV from the couch. |  |  |  |  |
| 17. The glasses have GPS so your loved ones can easily locate you. Example: If your loved one is not able to contact you and is worried about you, they can go on their phone to see where your glasses are, and therefore, where you are. |  |  |  |  |

**Of all the functions above: list the 3 functions you liked best.** *Evaluator: repeat each function.*

1st best: _______________________________________

2nd best: _______________________________________

3rd best: _______________________________________

**If a set of glasses had the three functions listed above, do you do you think you would actually wear them? Mark an X.**

_____ Yes

_____ No

_____ Only if they look and feel like normal glasses.

_____ For short periods at a time or during certain situations.

**Which aspect of smart glasses would cause you to actually wear the smart glasses? Rank from 1-4.**

____The look. The smart glasses would need to be fashionable (not bulky or odd looking) for me to wear them.

____ Their functionality. The smart glasses would need to drastically improve my quality of life for me to wear them.

____The battery life. The smart glasses battery would need to last at least a full day.

___ Waterproof. I don’t want to worry about the glasses in the rain.
