# Supplementary material for: Smart Glasses for Older Adults With Cognitive Impairment: Explanatory Mixed Methods Study
Source: JMIR Aging. 2026 Apr 27;9:e81840. doi: 10.2196/81840 (PMC13119387; doi:10.2196/81840)
Supplement: Multimedia Appendix 2 [file aging-v9-e81840-s002.docx]

Codebook

| **Codes & Sub-Codes** | **Frequency** |
| --- | --- |
| Code System | 288 |
| Meta Advantages | 4 |
| Translate | 2 |
| Other Functions | 6 |
| GPS | 4 |
| Answering Questions | 1 |
| Reminders | 8 |
| Aesthetics | 8 |
| Case | 4 |
| Responds Well/ Ease | 11 |
| Comfort | 10 |
| Camera | 7 |
| Meta Disadvantages | 15 |
| Alexa Advantages | 0 |
| Other Functions | 2 |
| Other Devices or GPS | 5 |
| Comfort | 7 |
| Reminders | 10 |
| Calls | 2 |
| Answering Questions | 14 |
| Aesethics | 5 |
| Alexa Disadvantages | 0 |
| Slow on Uptake | 9 |
| Use of Phone Rather Than Audio | 1 |
| Lack of Camera | 8 |
| Comfort | 3 |
| Vuzix Advantages | 0 |
| Interesting/Novelty | 3 |
| Apps | 7 |
| Touch or Voice | 4 |
| Valued for Specific Situations | 3 |
| Vuzix Disadvantages | 0 |
| Privacy Concerns/Conspicuous Nature | 3 |
| Little Voice Commands | 1 |
| Projection Difficult to See or Distracting | 11 |
| Name | 2 |
| Similarity with Phone or Computer | 2 |
| Clunky | 11 |
| Difficult to Navigate, Complicated, Sensitive | 20 |
| “Best” Smart Glasses | 14 |
| Most Valued Smart Glasses Functions | 14 |
| Functions You Wish Smart Glasses Could Do | 13 |
| Overall Smart Glasses Thoughts | 21 |
| Overall Smart Glasses and Memory | 13 |
