# Supplementary material for: Smart Glasses for Older Adults With Cognitive Impairment: Explanatory Mixed Methods Study
Source: JMIR Aging. 2026 Apr 27;9:e81840. doi: 10.2196/81840 (PMC13119387; doi:10.2196/81840)
Supplement: Multimedia Appendix 3 [file aging-v9-e81840-s003.docx]

Additional Participant Quotations

| **Theme** | **Participant Number** | **Quotation** |
| --- | --- | --- |
| Ray-Ban Meta | 24 | I liked everything about these glasses. It answers the questions I ask clearly and quickly. |
|  | 11 | I liked everything about them. I like the way they felt. I liked the size and shape of them. |
|  | 8 | I like the location of where I am is more accurate. Because I have dementia, there are times I'm not sure where I am. It is very scary. I have a GPS on my phone so I can find the quickest way to walk home. So this would be great for people with glasses. If I'm walking along with my wheels and I forget where I am it's good to know [referring to hands-free functionality]. |
| Alexa Echo Frames | 14 | Alexa seems to be a little slower than the first one [Meta]. It seems like you have to repeat a little bit more than the first. |
|  | 17 | I prefer the Meta. These did everything the Meta could do but take a picture and describe an item and I like that function. If I'm going to get glasses like this, I'll spend the extra 50-100 for the camera since it is available. If I get the technology, I want it all. |
|  | 8 | They are really handy. So many things I need to pull my phone out to do… I can just do that without having to pull something out. Like checking a shopping list. |
| Vuzix Blade 2 | 11 | It does have a lot of different applications. However, to me, because I'm not computer savvy like some folks are, I'm not prehistoric but I'm not 100% in the 21st century, I can't do lots of things on my phone. While it has a lot of good applications, for the likes of me, it might be a little too complex. |
|  | 2 | But other than that, I'm not the advanced person. It's hard to remember, "this finger, this side." It's too much to remember. I like the other ones because I can just talk right to it. |
|  | 7 | Maybe it was just my voice and not responding right away, or maybe I wasn’t pronouncing the word correctly [Vuzix], but I know it was sensitive because you were commanding it for example when you said “camera.” |
|  | 11 | I would worry if I'm moving, would it [the projection] be too much of a distraction. |
| Preferred Smart Glasses and Functions | 9 | Meta. Whatever you do, they are very comfortable. They are light weight. And they respond to you very quickly. You really don't need to do too much to it for it to cooperate. And it feeds back fast. They do everything for you. You don't have to ask for much. They are so easy to handle. It's easy to function with them. Some people, if you have to repeat over and over, they get frustrated. But for these, you don't. |
|  | 14 | Meta. Because it seemed easy to control. Easy to talk with. It picks up very well. It gave me straight answers very quickly and I like that about them. |
|  | 23 | Meta. Because they have the camera. It was easy. And you can charge in the case. And they don't have all that stuff up on the screen. They just seem simpler to me. I'm 65 so it helps. If Alexa had a camera, I still would like Meta. It was easier for Meta to understand me. I really like how they can remind me to take my medications or where I put my keys. Those are really good points at my age. |
|  | 7 | For me, being my age and can't remember things, it’s the reminders. That’s very helpful. Every day, I can't find something and remember where I put it. Today, it was my glasses I couldn’t find. Yesterday it was my pen. So reminders. That’s a big plus for me. |
| Functions Smart Glasses Should Do | 2 | They do everything. There's nothing that they can't do. The thing is… you have to charge them and I'm on a trip… I have to find where to charge my glasses. What if I'm gone longer than 8 hours? I like how you can charge the Meta in a case. |
|  | 24 | Say I'm at the bustop and [the glasses] say "here comes my bus," and actually see my bus, other than just reading off the time. If it could be connected to more things. |
|  | 8 | If it could zoom in to do street signs or hazards, or seeing my crochet close up...that would definitely be something I'd want. As of this point, none of them have that. |
|  | 3 | I don't know how it would do walking through the store so you don't back track. It would be nice if it could navigate through a store and group food items based on where you find them. |

Note: Additional quotations for theme “Broad Smart Glasses Concerns” omitted due to limited data.
